# Melatonin Engineered Adipose-Derived Biomimetic Nanovesicles Regulate Mitochondrial Functions and Promote Myocardial Repair in Myocardial Infarction

**DOI:** 10.3389/fcvm.2022.789203

**Published:** 2022-03-23

**Authors:** Yang Zhang, Ning Yang, Xu Huang, Yan Zhu, Shan Gao, Zhongyang Liu, Feng Cao, Yabin Wang

**Affiliations:** ^1^Department of Cardiology, The Second Medical Center and National Clinical Research Center for Geriatric Diseases, Chinese People's Liberation Army General Hospital and Medical School of Chinese People's Liberation Army, Beijing, China; ^2^Department of Cardiology, Zhongda Hospital, School of Medicine, Southeast University, Nanjing, China; ^3^Nankai University School of Medicine, Nankai University, Tianjin, China; ^4^Department of Orthopedics, The Fourth Medical Center of Chinese PLA General Hospital, Beijing, China

**Keywords:** myocardial infarction, melatonin, biomimetic nanovesicles, mitochondrial function, reactive oxygen species

## Abstract

Myocardial infarction (MI), one type of ischemic heart disease, is a major cause of disability and mortality worldwide. Currently, extracellular vesicles (EVs) derived from adipose-derived stem cells (ADSC) have been proven to be a potentially promising therapeutic treatment for MI. However, the inconvenience of isolation, the low productivity, and the high cost of EVs greatly limits their application in clinic. In this study, we constructed novel biomimetic ADSC-derived nanovesicles (ADSC NVs) to achieve cell-free therapy for MI. Here, we firstly developed a novel Mel@NVs delivery system consisting of engineered ADSC NVs with melatonin (Mel). Then, the characterization and properties of Mel@NVs were performed. The effect of Mel@NVs on cellular oxidative stress and myocardial infarction repair was conducted. The results showed that Mel@NVs treatment under ischemia mimic condition reduced cell apoptosis from 42.59 ± 2.69% to 13.88 ± 1.77%. Moreover, this novel engineered Mel@NVs could ameliorate excessive ROS generation, promote microvessel formation, and attenuate cardiac fibrosis, which further alleviates mitochondrial dysfunction and finally enhance myocardial repair. Hence, the engineered NVs show a potential strategy for MI therapy.

## Introduction

Myocardial infarction (MI), one type of ischemic heart disease (IHD), is the leading cause of disability and mortality worldwide (1). Myocardial injury is the most important factor which results in MI. Evidence has demonstrated that oxidative stress is an important pathophysiological mechanism of myocardial injury, and the production of excessive reactive oxygen species (ROS) can impair cell function and vitality (2, 3). Therefore, improving the survival rate of cardiomyocytes and inhibiting the production and accumulation of reactive oxygen species may be essential to improve the prognosis of ischemic diseases. Recent studies have shown that the application of antioxidant drugs could attenuate oxidative stress efficiently. Researchers have revealed that melatonin can resist oxidative stress damage in a variety of ways, including direct detoxification and indirect enzymatic hydrolysis of reactive oxygen species (4–7). In fact, melatonin is an indole heterocyclic compound secreted by the pineal gland (8, 9). For its antioxidative purpose to use in diseases, melatonin was also known as the antioxidant drug because of its efficacy on myocardial injury, which has been verified by several studies (10, 11). However, the half-life of the drug in the body is relatively low, which affects the actual therapeutic effect and causes side effects. Therefore, various nano-delivery carriers have been developed to carry drugs and deliver them to the injured site (12, 13). Unfortunately, the application of nanocarriers for drug delivery is limited due to their immunogenicity and toxicity, low loading capacity, and biodistribution problems (14, 15).

Recently, considerable progress has been concentrated on EVs. EVs are natural microvesicles secreted by many cell types with excellent biocompatibility, low cytotoxicity, immune inertness, and long-term circulation capabilities (16, 17). Due to their nano-scale size and excellent biocompatibility, EVs have been considered as natural carriers used in drug delivery applications (18, 19). Compared with commonly used synthetic carriers, the high biocompatibility of EVs makes them an ideal choice for drug delivery without causing adverse pro-inflammatory and immune responses. In addition to its unique characteristics, EVs are also enriched with specific components such as proteins, mRNAs, and miRNAs from parent cells, thereby playing critical roles in various physiological and pathological processes to transplant the natural biological functions of original cells, for example, cell proliferation, differentiation, and virus spreading, which can be directly used to treat many diseases (20, 21). Moreover, EVs derived from mesenchymal stem cells (MSC), such as bone marrow mesenchymal stem cells (BMMSC) and ADSC, have the capacity to enhance heart function after MI because EVs are rich in miRNAs (22, 23). MSCs derived EVs' miR-143-3p could reduce cardiomyocyte apoptosis by inhibiting chk2-beclin2 pathway to regulate autophagy (24). Meanwhile, ADSC-derived EVs overexpressing in microRNAs were studied to observe whether they have a better protective effect on MI, including miR-126 and miR-146a (25, 26). Therefore, EVs derived from adipose-derived mesenchymal stem cells not only have their own therapeutic effects but also have the advantages of rich sources and convenient access (27). However, the current extraction and purification of EVs and production efficiency have certain limitations, which hinder the possibility of EVs being used in clinical treatment. Recently, EVs mimicking nanovesicles (NVs), which have similar size and composition to EVs, have been harvested by continuously extruding cells through a microfilter (28, 29). EVs obtained by extruding the cells can not only retain the biological factors of the stem cells themselves but also greatly increase the productivity of the vesicles.

In this study, we constructed novel biomimetic ADSC-derived nanovesicles (ADSC NVs) to achieve cell-free therapy for MI. Here, we firstly developed a novel system consisting of engineered ADSC NVs with melatonin (Mel). NVs with controllable dimensions and high yield were harvested by continuously extruding ADSCs through filters with different pore sizes. Then the melatonin was loaded into the NVs by sonication to fabricate melatonin engineered NVs (Mel@NVs). Next, Mel@NVs was conducted to test its cardiac protective effect on cells and MI mice model both *in vitro* and *in vivo*, respectively. The ROS levels, apoptotic rate, histological morphologies, cardiac functions, and neovascularization were analyzed in this study.

## Materials and Methods

### Animals

C57BL/6j male mice (Adult, *n* = 40, 8 weeks old, 22–25 g, specific-pathogen-free) were obtained from SPF Biotechnology Co., Ltd. (Beijing, China). All operations were conducted under the Guidance of National Institutes of Health for the Care and Use of Laboratory Animals and acclaimed by the Ethics Committee of the Chinese People's Liberation Army General Hospital.

### Animals Grouping and Myocardial Infarction Model

The total animals were randomly divided into four groups (*n* = 10 each): (i) Sham surgery group (Sham group); (ii) MI + PBS group (MI group); (iii) MI + NVs group (NVs group); (iv) MI+Mel@NVs (Mel@NVs group). The ligation of the left anterior descending (LAD) artery was performed to establish the myocardial infarction (MI) model. Finally, mice were anesthetized by inhalation of isoflurane and received mechanical ventilation. LAD artery was received permanent ligation with a 6-0 silk suture after left thoracotomy. When the left ventricle (LV) had pale discoloration and an elevation of the ST segment on electrocardiographic (ECG) was recorded, it is confirmed that the vessel was successfully occluded. Mice in the sham-operated group were only performed thoracotomy without ligation. Then, NVs and Mel@NVs in 20 μl PBS were injected intramyocardially at three various portions in the area of the infarcted myocardium with a 30-gauge needle. MI group animals were injected with the same volume of PBS for control.

### Cell Culture

Adipose-derived stem cells (ADSC) were obtained by the Department of Orthopedics, Peking University People's Hospital (acquired from Cellular Biomedicine Group). ADSCs were cultured in Growth Medium for Human ADSCs (Cyagen Biosciences, Suzhou, China). The H9C2 cell line was obtained from Procell Life Science & Technology (Wuhan, China). This type of cell was cultured in Dulbecco's Modified Eagle's Medium (DMEM, Hyclone, Logan, Utah, USA) added with 10% fetal bovine serum (FBS, Gibco, USA) and 1% antibiotic (Invitrogen, CA). All assays were expressed in a humidified environment full with 5% CO_2_ at 37°C.

### Establishment of H/SD Cell Models *in vitro*

The hypoxia/serum deprivation (H/SD) cell model was established by hypoxia/serum deprivation. In short, H9C2 cells were cultured in Hanks buffer for 24 h and cultured in a 37°C hypoxic environment (1% O_2_, 5% CO_2_, and 94% N_2_). The control cells group were normally cultured in 95% air and 5% CO_2_ for an equal time.

### Characterization of ADSCs

The surface specific markers of ADSC were analyzed by flow cytometry (FACS-Calibur, BD Biosciences, California, USA). The adipogenic, osteogenic, and chondrogenesis differentiation of ADSCs were conducted for confirming ADSCs' multi-cell differentiation ability. The morphologic images of cells were obtained using an optical microscope (Olympus Corporation, Tokyo, Japan).

### Preparation of NVs

ADSCs were washed three times with cold PBS and harvested by cell scrapers and then suspended in PBS with a concentration of at least 1 × 10^6^ cells/ml. The cell suspensions were sequentially extruded through 1 μm, 400 nm, and 200 nm polycarbonate membrane (Whatman Inc, USA) using a mini-extruder (Avanti Polar Lipids) five to six times. Here, we defined the extruded vesicles as nanovesicles (NVs). The harvested NVs were diluted in PBS and stored at −80°C.

### Characterization of NVs

The zeta-potential, size distribution and concentration of NVs were measured using ZetaView (Particle Metrix, Germany). The morphologic images of NVs were observed via transmission electron microscopy (TEM, JEM-2000EX, Japan). The number of proteins in the NVs was analyzed using total proteins using the BCA Protein Assay Kit (Solarbio, China). The number of specific EV proteins included were observed by western blot.

### Melatonin Loading Into NVs

Melatonin was loaded into the NVs (1 mL) and the fluid solution was conducted under ultrasonic treatment (30) (20% amplitude, 7 cycles, 15 s on/off, 180 s duration, 120 s cooling period between each cycle). After sonication, the mixture was then cultured at 37°C for 240 min to allow for recovery of the nanovesicle membrane to form Mel@NVs. Mel@NVs were obtained by ultrafiltration and excessive free melatonin was removed from the supernatant. The loading efficiency of melatonin encapsulated into nanovesicles was measured via high-performance liquid chromatography (HPLC, LC-20AD, Shimadzu, Japan).

### Cellular Uptake of Mel@NVs

For observing nanovesicles tracing in H9C2 cells, nanovesicles were labeled with PKH26 (Sigma Aldrich, USA) by dying with the staining applied according to the manufacturer'sinstructions. H9C2 cells incubated in a confocal cell dish were cultured with PKH26-dyed nanovesicles for 4 h and washed with PBS. Then, cells were treated with 4% paraformaldehyde for 10 min and rinsed with PBS. After rinsing, 0.5% triton X-100 was used to permeabilize cells for 300 s, and then PBS was used to wash cells. Cells were subsequently stained with FITC-phalloidin (Solarbio, China) for 30 min and rinsed with PBS. Finally, cells nuclei were labeled with DAPI (Beyotime, China). Cellular uptake of the nanovesicles was obtained by using a confocal microscope (Nikon, Japan).

### Detection of Cellular and Mitochondrial ROS

The quantity of cellular ROS production was detected by dihydroethidium (DHE, Beyotime, China) staining in line with the manufacturer's instructions. In brief, cells were cultured with 1 mM DHE probe for 30 min in darkness at 37°C, and then rinsed with PBS three times and subsequently observed. The quantity of mitochondrial ROS production was determined by mitochondrial superoxide indicator (MitoSOX, Invitrogen) corresponding to the instructions. In short, cells were rinsed twice with PBS and loaded with the probes (4 μM) for 10 min in the dark atmosphere at 37°C. Then cells were rinsed twice and stained with DAPI. The images were then captured by a confocal microscope (Nikon, Japan). The fluorescence of DHE or mitoSOX was analyzed according to previous studies (21, 31, 32). In general, these obtained fluorescence images were run and calculated via Image J software.

### Cytokines Measurement

Cell supernatant was collected in cold conditions. Enzyme-linked immunosorbent assay (ELISA) was performed in accordance with the manufacturer's instructions to measure the level of anti-inflammatory cytokines (TGF-β and IL-10) and pro-inflammatory cytokines (IL-6 and TNF-α) (Xi-Tang, Shanghai, China).

### Western Blot

Cells were rinsed with pre-cooling PBS three times and scraped and then lysed with RIPA (Solarbio, China) containing 1 mM phenylmethylsulfonyl fluoride (PMSF, Beyotime) on ice for 10 min. After centrifugation at 12,000 rpm for 10 min at 4°C, the supernatant was obtained and the quantity of protein was analyzed by BCA Protein Assay Kit (Solarbio, China). The protein extract was separated by 12% SDS-PAGE at 150 V for 60 min then transferred to the PVDF membrane under 200 mA current for 60 min. Under blocking, the membrane was cultured with the primary antibody for 8 h at 4°C, washed with TBST, and treated with a suitable secondary antibody for 60 min at room temperature and rinsed with TBST. The immunoblotting was observed using an ECL substrate kit (Millipore, Germany). The using antibodies included Bax antibody (1:1,000, Cell Signaling Technology (CST), USA), Bcl-2 antibody (1:1,000, CST, USA), and β-actin antibody (1:5,000, Proteintech, USA).

### Flow Cytometry Assay

Cells were harvested and blown for 5 min to obtain a single-cell suspension, washed with cold PBS three times. Then, cells were cultured with Annexin V-FITC/binding buffer solution for 30 min in the dark atmosphere. Finally, the mixture was incubated with PI buffer for 10 min in line with the manufacturer's instructions (BD Biosciences, USA). The apoptosis and necrosis rate of cells were analyzed by flow cytometry.

### Echocardiogram Detection

All mice were examined by echocardiography 4 weeks after the operation. After the mice were inhalation-anesthetized, heart function was measured by echocardiography. M-mode images and left ventricular ejection fraction (LVEF) were measured. All operations and analyses were performed by an experienced researcher who was unaware of the experimental group.

### Heart Tissue Immunofluorescence Staining

The left ventricle including the MI area was embedded in paraffin. Hematoxylin-Eosin (H&E) staining and Masson staining were performed separately. An optical microscope (Olympus, Japan) was used to analyze the morphological changes of the nucleus and cytoplasm around the MI marginal zone in H&E staining. Masson staining observed collagen scar tissue and myocardial fibrosis at 4 weeks postoperatively. Immunofluorescence staining with anti-CD31 antibody (Abcam, USA) was used to examine the formation of new blood vessels in the sections. The density of microvessels was analyzed as the number of vessels per field under microscope according to Lin's previous study (11).

### Statistical Analysis

All experimental data were expressed as mean ± SEM. Student's *t*-test was used for comparison between two groups, and one-way ANOVA was performed for multiple comparisons among more than two groups. These data were analyzed and processed by GraphPad Prism 9.0 (GraphPad Software, San Diego, California). When the *P*-value was < 0.05, the differences were considered statistically significant.

## Results

### Characterization of ADSCs and ADSC-Derived NVs

**Figure 1:**
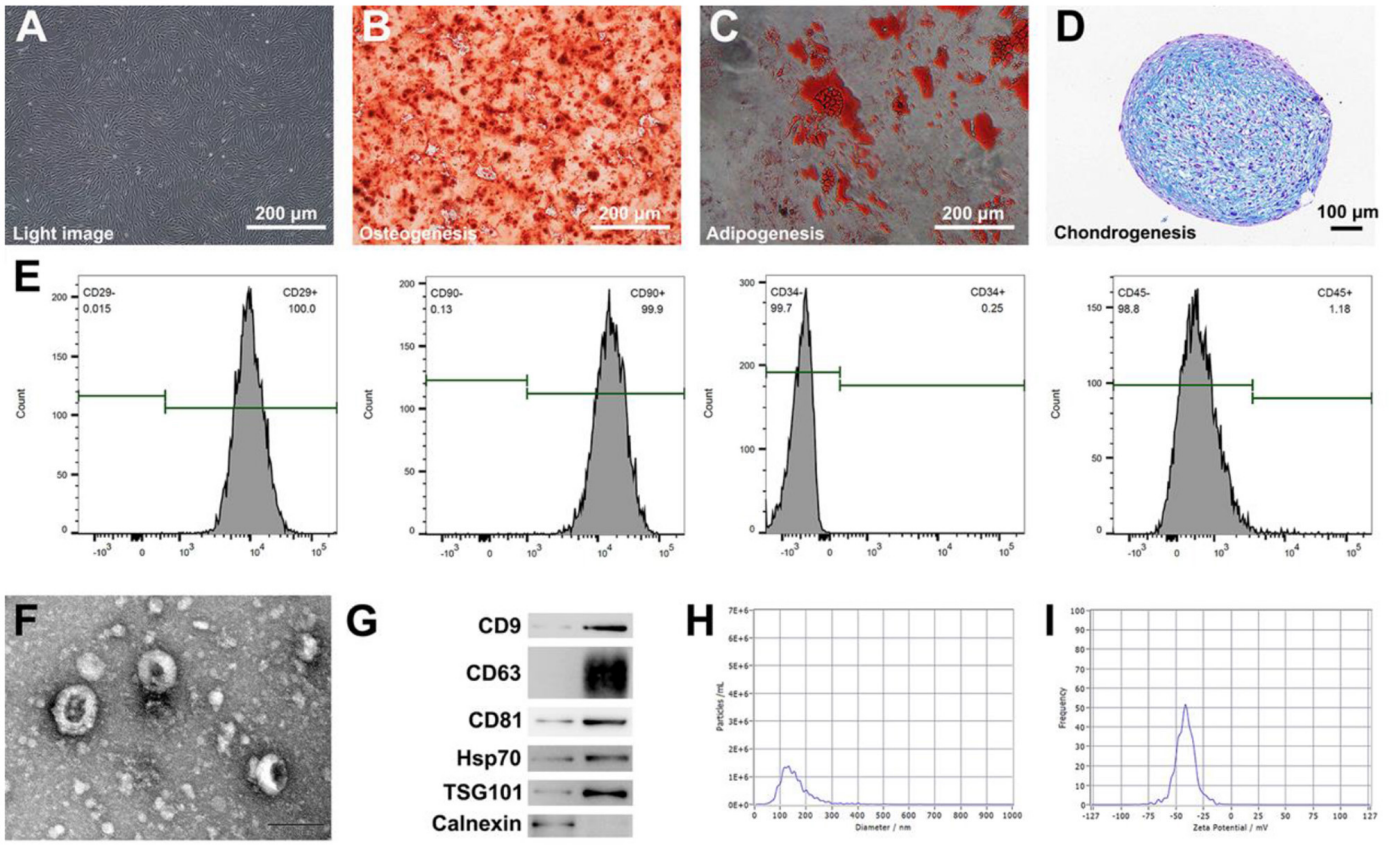
Characterization of ADSCs and ADSC-derived biomimetic nanovesicles. **(A)** The morphology of ADSCs by using a microscope. Scale bar = 200 μm. **(B–D)** Osteogenic, adipogenic, and chondrogenic differentiation of ADSCs. Scale bar = 200 μm. **(E)** Analysis of flow cytometry indicating surface markers of ADSCs. **(F)** The characteristic image of ADSC-derived biomimetic nanovesicles under TEM. Scale bar = 100 nm. **(G)** Western blot analysis of nanovesicles' positive markers including CD9, CD63, CD81, Hsp70, and TSG101. Calnexin was used as the negative control. **(H,I)** Size distribution and zeta potential of nanovesicles.

The morphology of ADSCs was observed using an optical microscope (Figure 1A). The multipotential differentiation properties of ADSCs were confirmed through osteogenesis, adipogenesis, and chondrogenesis experiments in response to appropriate inducement (Figures 1B–D). To gain further understanding of ADSCs, the immunophenotype of the ADSCs was detected by flow cytometry. The positive markers were CD90 and CD29, meanwhile, the negative markers were CD34 and CD45 (Figure 1E); these features confirmed that the cells were ADSCs. Next, we prepared NVs by continuously extruding ADSCs. The morphological image of nanovesicles derived from ADSCs was then observed by TEM, and the obtained nano-vesicles showed a morphology similar to typical exosomes (Figure 1F). Western blot analysis demonstrated that NVs expressed exosome-specific markers, namely TSG101, CD9, CD63, CD81, and Hsp70. In contrast, calnexin was not observed in isolated exosomes (Figure 1G). Moreover, the average size of NVs was 141.7 ± 59.2 nm (Figure 1H), and the value of zeta potential in NVs was −41.1 mV (Figure 1I).

### Preparation and Characterization of Mel@NVs

**Figure 2:**
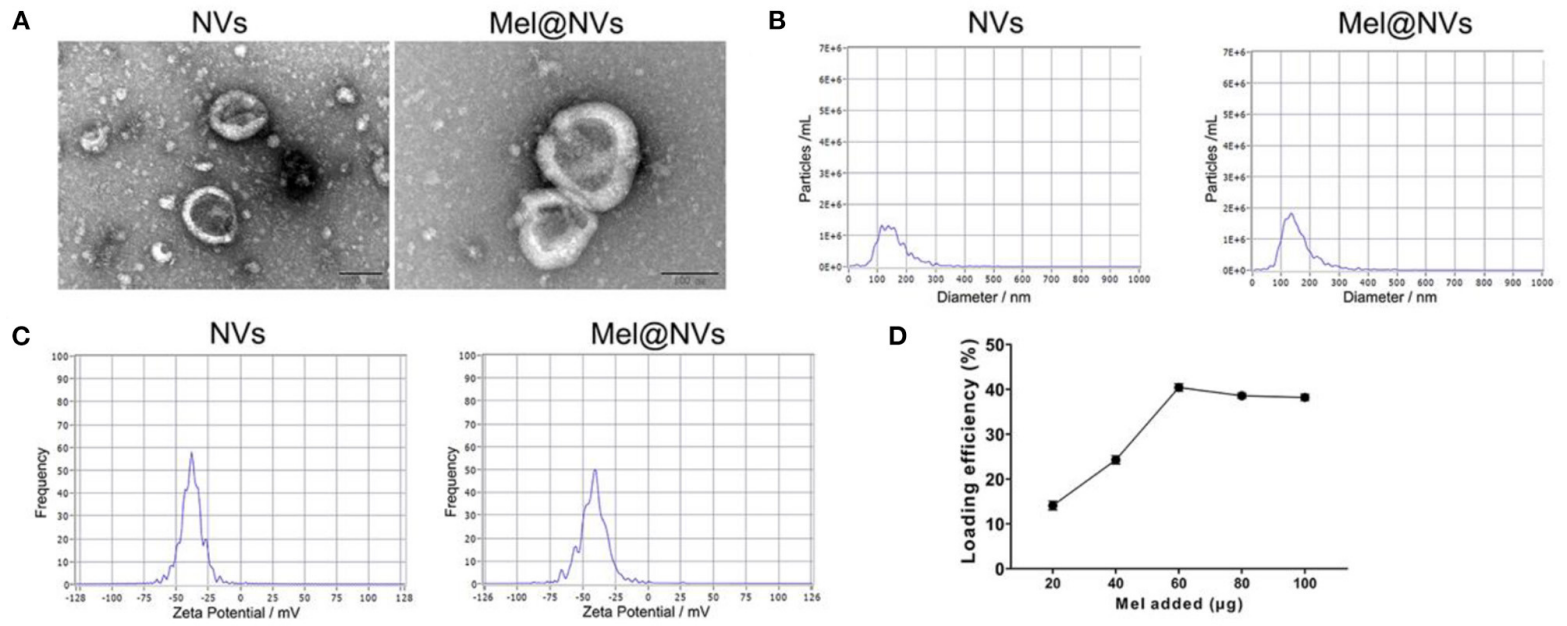
Preparation and characterization of Mel@NVs. **(A)** The characteristic images of NVs and Mel@NVs via TEM. Scale bar = 100 nm. **(B,C)** Size distribution and zeta potential of Mel@NVs. **(D)** HPLC analysis of loading efficiency. A different amount of melatonin was added into NVs (10 μg). The loading was sonicated when 40 μg melatonin was added. The results represented the mean ± standard deviation (*n* = 3).

Next, we loaded melatonin into NVs by sonication. Figure 2A showed that the morphology of Mel@NVs was similar to that of NVs, with a characteristic cup-shaped structure. The average size of Mel@NVs was 142.9 ± 60.4 nm, which was compared to that of NVs (144.6 ± 59.3 nm) (Figure 2B). Meanwhile, the values of zeta potential (Figure 2C) in Mel@NVs and NVs were −41.5 and −37.9 mV, respectively. Moreover, high-performance liquid chromatography (HPLC) analysis showed that the melatonin was loaded successfully, and the loading capacity of Mel was 40.42% (Figure 2D). These results indicated that there were no big changes in the surface properties of Mel@NVs after loading melatonin into NVs.

### Cellular Uptake and Cardio-Protective Effects of Mel@NVs in H9C2 Cells

**Figure 3:**
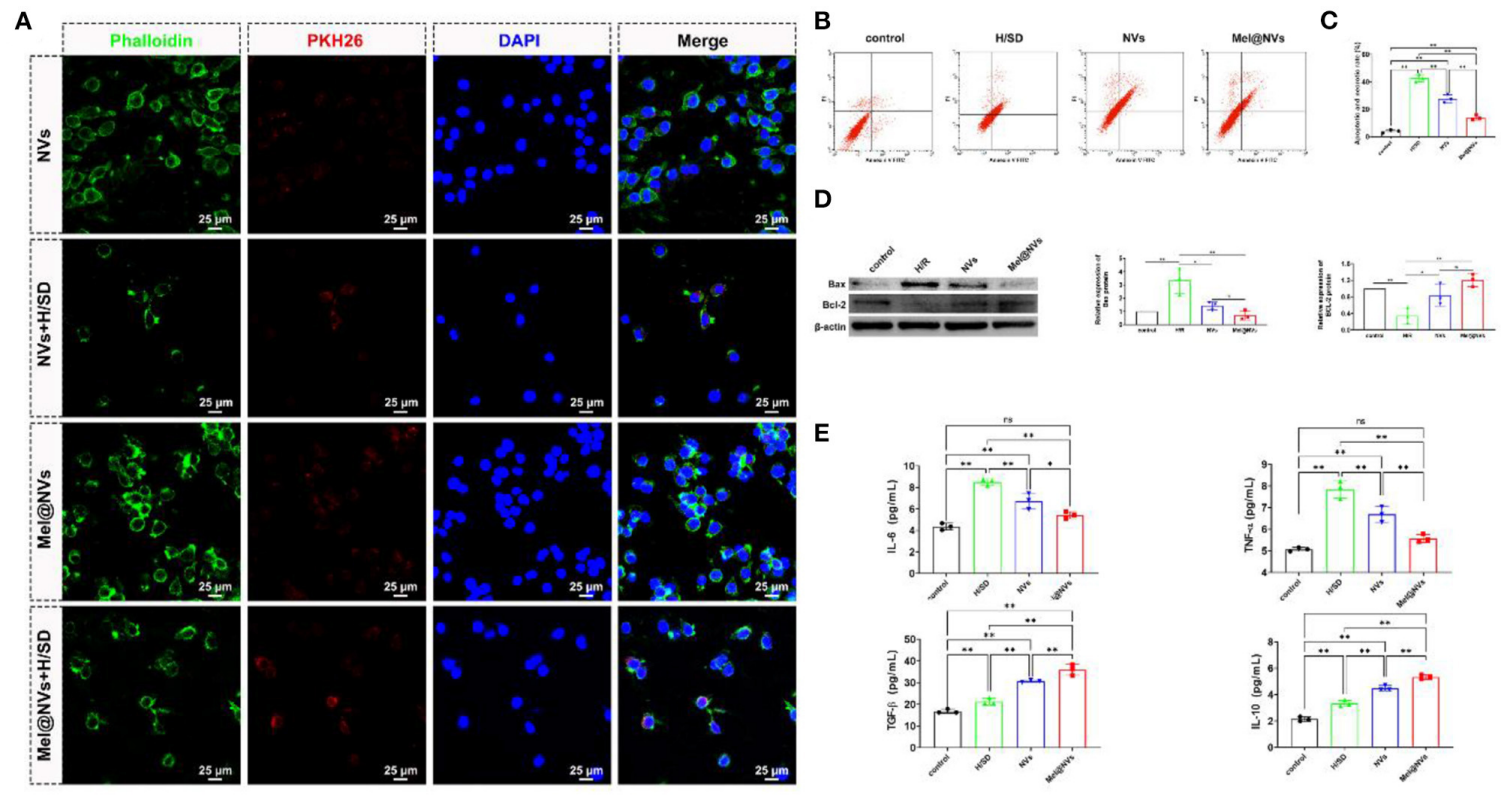
Cellular uptake and cardioprotective effects of Mel@NVs in H9C2 cells. **(A)** Representative images of NVs and Mel@NVs taken up by H9C2 cells in control and H/SD conditions. **(B)** The apoptotic rate of annexin-V/PI staining in different conditions by using flow cytometric detection. **(C)** The analysis of the quantity of apoptotic and necrotic cells by flow cytometry (**p < 0.01). **(D)** Detection of apoptosis-related proteins using Western blotting test. **(E)** The amount of anti-inflammatory and pro-inflammatory cytokines using ELISA in H9C2 cells with different treatments (*p < 0.05; **p < 0.01; ns, not significant. *n* = 3).

In order to observe the uptake of NVs at a cellular level, we labeled NVs and Mel@NVs with red-fluorescent protein PKH26, and labeled the cell cytoskeleton with phalloidin (green color). H9C2 cells were observed under fluorescence microscopy after being incubated PKH26-stained NVs and Mel@NVs (Figure 3A). The fluorescence images showed that PKH26-labeled NVs and Mel@NVs were taken up by H9C2 cells and scattered around the nucleus. Even after 24 h of hypoxia treatment, Mel@NVs can still be taken up by cardiomyocytes. Flow cytometry analysis quantitatively detected the rates of apoptosis and necrosis in the four groups. The apoptotic and necrotic rate of cardiomyocytes was significantly reduced in the Mel@NVs treatment group (13.88 ± 1.77%) compared to that in H/SD treatment group (42.59 ± 2.69%) and NVs treatment group (27.56 ± 3.04%). In order to analyze mitochondrial apoptotic effect, western blotting was performed to detect mitochondrial apoptosis-related proteins, such as Bax and Bcl-2. The western blot results demonstrated that Mel@NVs significantly down-regulated pro-apoptotic protein (Bax) and up-regulated anti-apoptotic protein (Bcl-2). Furthermore, the levels of anti-inflammatory factors (such as TGF-β and IL-10) and pro-inflammatory factors (such as IL-6 and TNF-α) were analyzed by ELISA assays. As shown in Figure 3, compared with the H/SD group, NVs and Mel@NVs groups both showed higher levels of anti-inflammatory cytokines, but the lower level of pro-inflammatory cytokines, indicating the effective anti-inflammatory properties of Mel@NVs on cardiomyocytes under ischemia mimic condition *in vitro*. These experiments showed that Mel@NVs have a better cytoprotective effect than NVs ascribed to the introduction of melatonin into NVs.

### The ROS Scavenge and Mitochondrial Functional Protection of Mel@NVs *in vitro*

**Figure 4:**
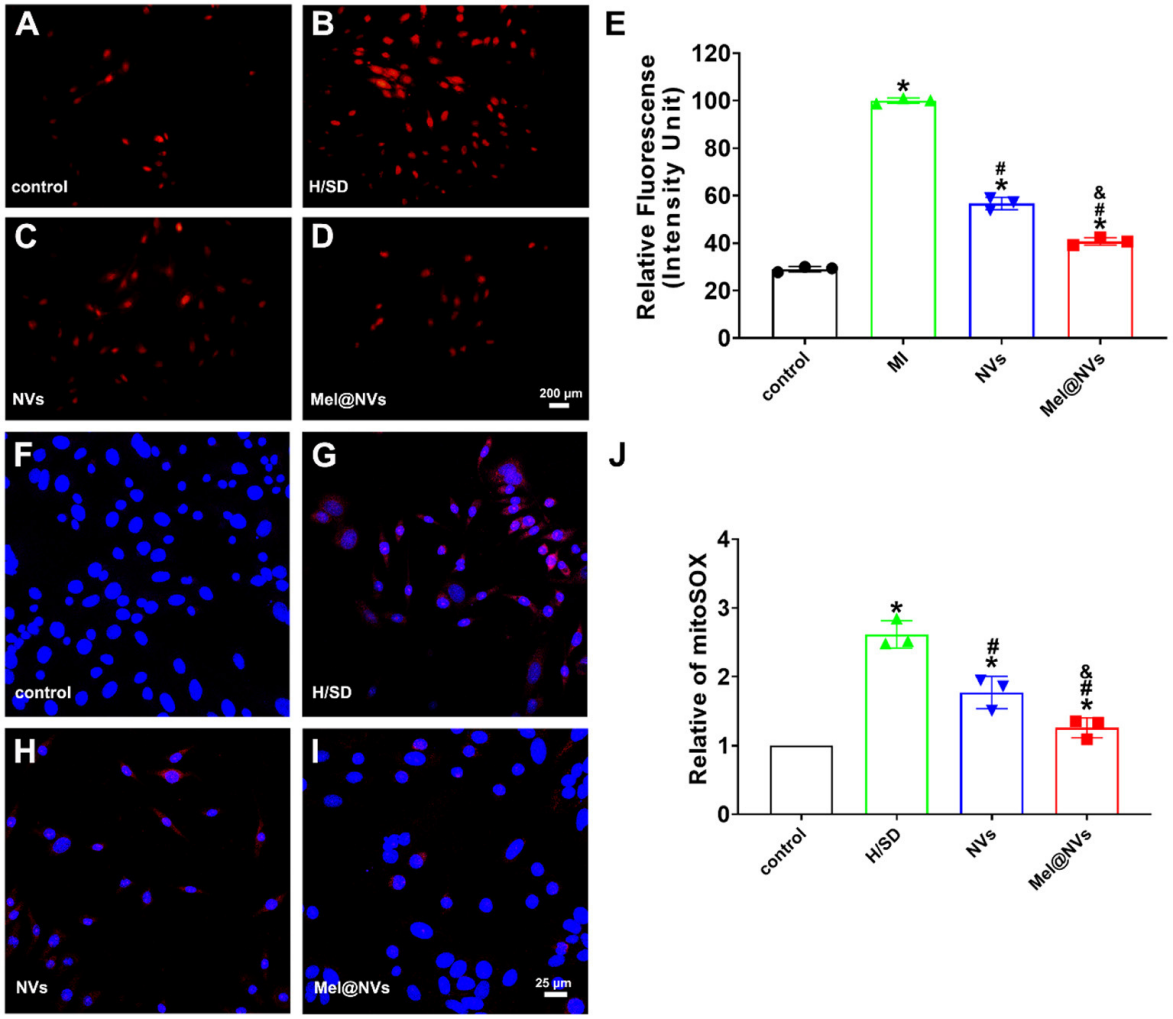
The effect of Mel@NVs on reactive oxygen species levels in H/SD condition. **(A–D)** The ROS levels in cells by using DHE probe and the images were obtained using fluorescence microscopy. Scale bar = 200 μm. **(E)** The quantification of the DHE fluorescence intensity is shown in a bar graph (*p < 0.05 v.s. control; #*p* < 0.05 v.s. H/SD treatment; &*p* < 0.05 v.s. NVs treatment). **(F–I)** The oxygen species levels in mitochondria were observed by using a Mito-SOX probe and the images were obtained using confocal laser scanning microscopy. Scale bar = 25 μm. **(J)** The quantification of Mito-SOX fluorescence intensity is detected in different assays (*p < 0.05 v.s. control; #*p* < 0.05 v.s. H/SD treatment; &*p* < 0.05 v.s. NVs treatment). The results represented the mean ± standard deviation (*n* = 3).

To observe whether Mel@NVs play a cardio-protective effect on scavenging ROS, DHE staining was used to detect ROS levels. Figures 4A–E showed that the red fluorescence intensity, which represents the level of ROS, was the lowest in the control group, and the highest in H/SD treatment group. The fluorescence intensity in the NVs treatment group was lower than that in H/SD group. Interestingly, Mel@NVs treatment group showed a significantly reduced intensity than NVs addition. Moreover, mitoSOX, a mitochondrial ROS detection probe, was used to observe the production of ROS in mitochondria in different treatment groups. Figures 4F–J showed that under H/SD exposure, the level of mitoSOX was the highest compared to other groups. After the introduction of Mel@NVs, the intensity of ROS was lower than that in the NVs treatment group. All the above data showed that Mel@NVs could reduce cellular and mitochondrial oxidative stress effects, which might be a novel cardioprotective drug platform for MI.

### Therapeutic Effects of Mel@NVs in MI Model

**Figure 5:**
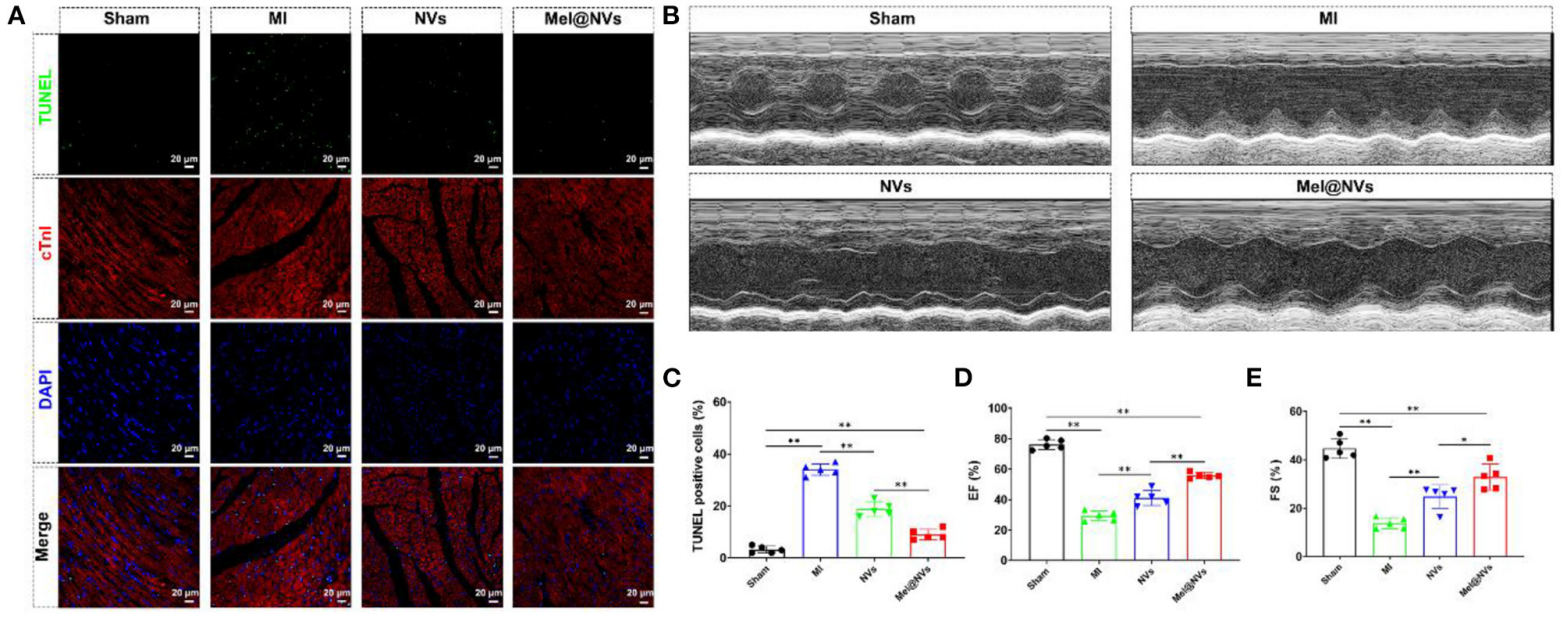
Apoptotic effect and cardiac function at 4 weeks after MI treatment in mice. **(A)** Representative fluorescence images of TUNEL-stained myocardial sections. The images showed TUNEL-positive cells (green color), myocardium stained with monoclonal antibody cTnI (red color), and total nuclei stained with DAPI (blue color). Scale bar = 20 μm. **(B)** M-mode echocardiography for heart function evaluation. **(C)** The quantification of representative TUNEL-positive apoptotic cells (**p < 0.01). The results represented the mean ± standard deviation (*n* = 5). **(D)** The ejection fraction rate (EF%) standing for heart function is presented as a bar graph (**p < 0.01). The results expressed the mean ± standard deviation (*n* = 5). **(E)** The fractional shortening (FS%) standing for heart function is represented as a bar graph (*p < 0.05; **p < 0.01). The results represented the mean ± standard deviation (*n* = 5).

**Figure 6:**
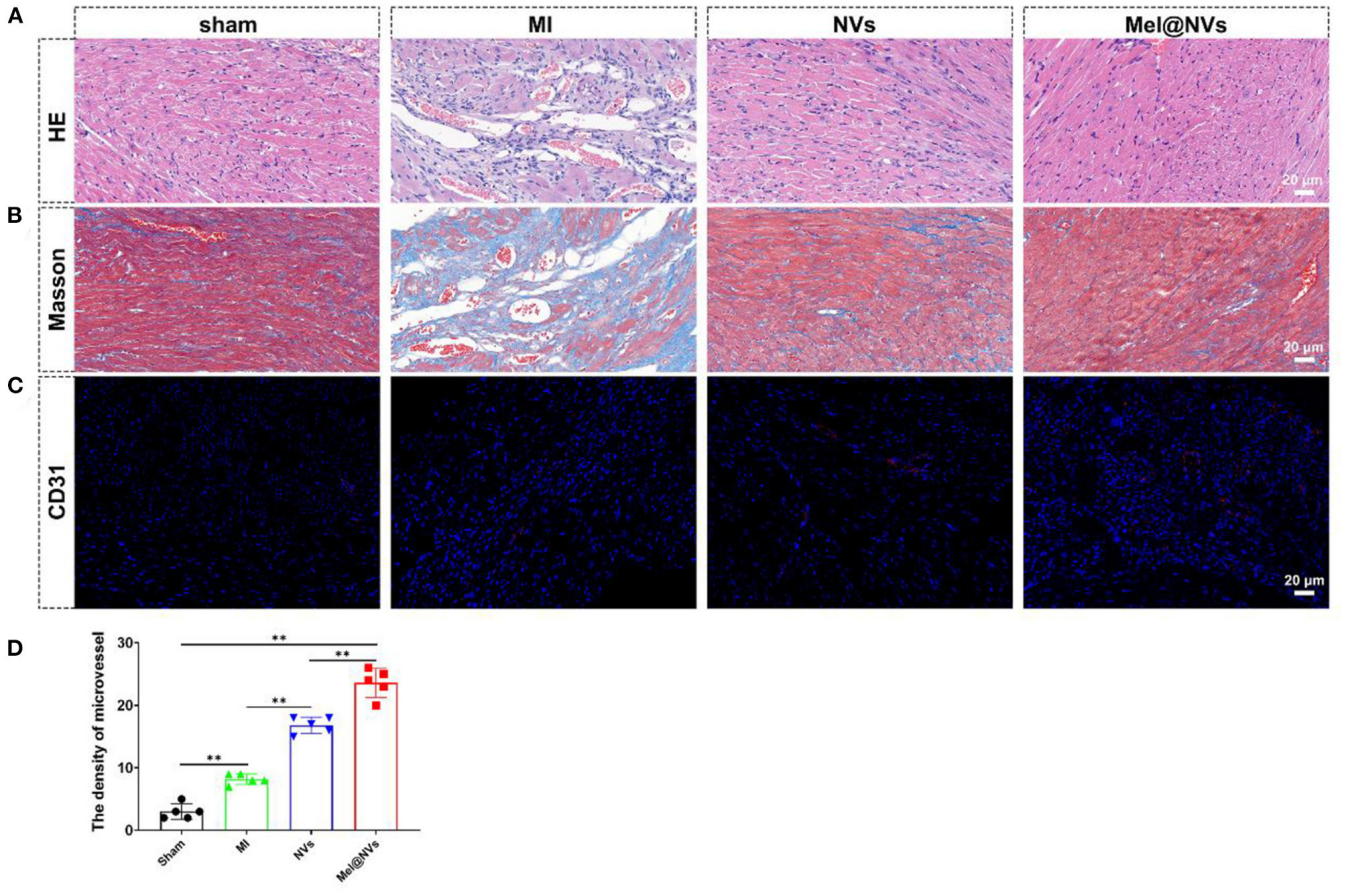
Histological evaluation of the effect of Mel@NVs in MI model at 4 weeks after MI in mice. **(A)** H&E staining in different groups. **(B)** Masson's trichrome staining in different groups. **(C)** Representative immunohistochemical dyed for CD31-positive cells in different groups (red color), and DAPI (blue color). **(D)** Quantitative analysis of micro-vessels in different groups (**p < 0.01). The results represented the mean ± standard deviation (*n* = 5).

In order to evaluate the potential cardioprotective effects of Mel@NVs *in vivo*, NVs and Mel@NVs were micro-injected directly into the myocardium of MI mice model. To confirm whether Mel@NVs inhibited cell apoptosis *in vivo*, TUNEL staining was used. As shown in Figure 5A, compared with the MI group, TUNEL-positive cells were significantly reduced in the heart sections of Mel@NVs injected-mice (Figure 5B). After 4 weeks, the cardiac function after myocardial infarction was measured by M-mode echocardiography (Figure 5C), and EF% and FS% were evaluated (Figures 5D,E). Compared with the MI group, the levels of EF% and FS% of mice injected with Mel@NVs increased significantly after MI. In addition, the reduction in myocardial remodeling and fibrosis assessed by H&E staining and Masson staining were observed among groups (Figures 6A,B), demonstrating that Mel@NVs treatment could reduce cardiomyocyte fibrosis. In addition, angiogenesis is an important factor that indicates the repair of ischemic myocardium. Therefore, we performed CD31-stained assay to observe the neovascularization. Compared with MI mice, Mel@NVs treatment significantly increased the expression level of CD31 (Figures 6C,D). According to the above experiments, Mel@NVs showed effective cardioprotection in infarcted hearts by reversing functional decompensation and pathological remodeling.

## Discussion

In this study, we first obtained nanovesicles derived from ADSC by continuous extrusion method, and then successfully constructed melatonin engineering biomimetic nanovesicles after sonication. Both *in vitro* and *in vivo* experiments have illustrated that Mel@NVs could reduce oxidative stress and improve myocardial injury repair effect.

Previous studies have shown that the obstructed vessels were unable to supply blood to the heart and then caused MI, leading to massive death of myocardial cells, followed by inflammation and oxidative stress (33, 34). The infarcted microenvironment full of inflammation and oxidative stress were the main reasons for the low survival rate of cardiomyocytes. The abnormal mitochondrial metabolism caused by excessive reactive oxygen species (ROS) severely affects the health of mitochondria in cardiometabolic diseases such as MI (35). Therefore, inhibiting oxidative stress has been proven to reduce cardiomyocyte apoptosis and alleviate cardiac dysfunction (36).

Melatonin has been reported to reduce cell injury in oxidative stress environments by neutralizing free produced radicals (37). This drug is anti-apoptotic, anti-inflammatory, and is widely used in many diseases, including ischemic heart disease (38). Previous literature has shown that melatonin can reduce the production and accumulation of ROS, maintain the structural integrity of mitochondria to protect mitochondria from ischemic damage (39). Moreover, melatonin can play a cardioprotective effect with its antioxidant activity and ability to inhibit neutrophils in myocardial tissue (40). However, its application in the treatment of MI has been limited because of the low half-life in the body. In our study, we used a simple but effective strategy to encapsulate melatonin into biomimetic stem cell-derived nanovesicles to fabricate a novel engineered Mel@NVs delivery system.

In recent years, extracellular vesicles (EVs) from various types of cells have been obtained and further applied in many diseases. EVs are natural membrane vesicles that participate in cell-to-cell communication (41). In addition to being loaded with complex cargo from parental cells, including proteins, mRNAs, microRNAs (miRNAs), and additional macromolecules, EVs have become attractive candidates for drug delivery applications for transferring their contents to recipient cells through endogenous uptake (42, 43). However, the current common method for extraction and purification of EVs is complicated and time-consuming, which limits their application in clinic. In this study, a serial-filters extrusion approach was conducted and NVs were obtained which possessed the morphologies and characteristic biomarkers of EVs, in line with previous studies. Next, Mel@NVs were designed by using a method of loading melatonin into nanovesicles via ultrasonic treatment. The results showed that Mel@NVs were successfully fabricated and the productivity of drug-loaded vesicles was higher.

In order to detect the effect of Mel@NVs on ROS, we used a hypoxia and serum deprivation (H/SD) environment, which could represent an oxidative stress condition. Mel@NVs could reverse the apoptotic rate after H/SD treatment, reduce pro-inflammatory factors, and promote anti-inflammatory cytokines, indicating that this novel Mel@NVs system could establish a protective milieu for cardiomyocytes under ischemia mimic condition *in vitro*. In addition, the DHE staining and mitoSOX experiments were performed and the results showed that the levels of ROS in cells and mitochondria were significantly reduced in the Mel@NVs treatment than that in NVs treatment. *In vivo* experiments showed that Mel@NVs treatment was able to reduce cardiomyocyte apoptosis and fibrosis, most importantly, promote the formation of microvessels, and finally improve cardiac functional recovery. Therefore, our strategy of combining melatonin and stem cell-derived NVs can not only inhibit oxidative stress and protect mitochondrial function through melatonin, but also use the vesicle's own protein and other nutritional factors to improve the microenvironment and ameliorate myocardial infarction.

In fact, previous studies have demonstrated that EVs not only possess the function of a drug carrier, but themselves also have anti-apoptotic effects used to treat animals in myocardial infarction inherited from their parental cells including ADSCs. EVs released by human cardiac progenitor cells have cardioprotective effects and improve cardiac function after myocardial infarction to the same extent as their parental cells. EVs derived from cardiac progenitor cells are rich in cardioprotective microRNAs, especially mir-146a-3p (44). MiRNAs are small, non-coding RNAs that regulate gene expression in a sequence-dependent manner (45). They play an important role in cell proliferation, differentiation, and even tumorigenesis and development (46). In recent years, studies have demonstrated that miRNAs are dysregulated in response to ischemic injury of the heart and actively contribute to cardiac remodeling after MI (47). There was a paper published in 2012 indicating that the therapeutic targeting of miR-15 in mice reduces infarct size and cardiac remodeling and enhances cardiac function in response to MI (48). Moreover, EVs of miR-126 overexpressing ADSCs reduce H9C2 cells damage by reducing the expression of inflammatory factors during hypoxia induction. Under hypoxic conditions, they can reduce the expression of fibrosis-related proteins in H9C2 cells and significantly promote the generation and migration of microvessels. It was confirmed that EVs rich in miR-126 can significantly reduce the myocardial injury area of infarction *in vivo* studies. These results demonstrated that ADSC-derived EVs overexpressing miR-126 could prevent myocardial injury by protecting cardiomyocytes from apoptosis, inflammation and fibrosis, and increasing angiogenesis (25). It was confirmed MSC-derived EVs can polarize M1 macrophages to M2 macrophages *in vivo* and *in vitro* experiments. MiRNA sequencing and bioinformatics analysis of MSC EVs suggest that miR-182 is a valuable candidate mediator for macrophage polarization. Reducing mir-182 in MSC EVs could partially weaken its regulation of macrophage polarization. Administration of MSC EVs to mice by intramyocardial injection after myocardial I/R reduced infarct area and alleviated Inflammatory levels in heart and serum. The results showed that MSC EVs changed the polarization state of macrophages through miR-182 and offered a novel method to reduce myocardial I/R injury in mice (49). MiRNAs could not only promote or inhibit cardiomyocyte death but also regulate angiogenesis after ischemia. MiRNAs that possess stem cell or progenitor cell-mediated cardioprotection can regulate cardiac regeneration so that they have great therapeutic potential in the treatment of acute myocardial infarction. For us, it is necessary to further explore the miRNAs that play a protective role against myocardial injury in stem cell-derived EVs and its underlying mechanism in the future.

Besides those above-mentioned EVs containing miRNAs possessing cardioprotection, the application of EVs as biomimetic drug carriers has become a significant research hotspot. EVs contain several desirable properties: the inherent ability to carry bioactive substances including proteins; immune tolerance with great biocompatibility; ideal stability in body fluids; natural targeting characteristics of cells; and the ability to cross biological barriers (41, 50). On the other hand, the tiny size of EVs provides favorable benefits for drug delivery, including reducing phagocytosis of circulating monocytes and passive accumulation to diseased tissues through imaged blood vessels (51). The unique characteristics of EVs promote the development of EVs based drug carriers in clinic. So far, no EV based therapies are available on the clinical application. Nevertheless, a few clinical trials were designed to explore the safety of EVs as drug delivery carriers. A phase I clinical trial (nct01294072) is currently being studied to explore the ability of plant-derived exosomes to deliver curcumin to normal and colonic tissues for the treatment of colon cancer. In another ongoing phase II clinical trial (nct01854866), the safety and efficacy of using tumor cell-derived exosomes as chemotherapeutic drug carriers in the treatment of malignant ascites. The above clinical trials have proved that autologous administration based on EVs as drug delivery carriers usually encompass no serious toxicity and adverse side effects, suggesting the safety and feasibility of this method. Biomimetic nanotechnology can create drug delivery systems similar to EVs. Extrusion technology can produce nanovesicles, which have the ability to produce vesicles more efficiently, and this method is highly biomimetic (52–54). In our study, the application of Mel@NVs has a significant therapeutic effect on infarcted myocardium *in vitro* and *in vivo*, further experimental studies are still needed in the future. The obstacle in the clinical application of NVs is the need for scalable EVs separation methods and more effective drug delivery methods for different therapies. Another challenge is to transform NVs from non-specific organ accumulation to targeted accumulation in desired tissues. Therefore, exploring new technological approaches to produce NVs with high targeting efficiency and safety will be our main research direction in the future.

In summary, our results showed that Mel@NVs protected the vitality of myocardial cells *in vitro* by reducing oxidative stress damage and apoptosis, and infarcted myocardial tissue *in vivo*. Through the use of the myocardial infarction model, it has been confirmed that the designed Mel@NVs play a therapeutic role in cardiac repair. Therefore, engineered nanovesicles will provide a potential strategy for the treatment of myocardial infarction.

## Data Availability Statement

The original contributions presented in the study are included in the article/supplementary material, further inquiries can be directed to the corresponding author/s.

## Ethics Statement

The animal study was reviewed and approved by The Institutional Animal Care and Use Committee of Chinese PLA General Hospital.

## Author Contributions

FC and YW conceived and designed the experiments. YZha, XH, and YZhu performed the experiments. YZha, XH, YZhu, SG, and ZL analyzed the data. YW and FC provided financial support and co-wrote the article. YW edited the manuscript. All authors contributed to the article and approved the submitted version.

## Funding

All authors are grateful to the Beijing Municipal Natural Science Foundation (7202189), National Nature Science Foundation of China (81530058, 81970443, 81671731, 81570272, 81571841, and 91939303), National Key Research Program of China (2016YFB0303303), NSFC Projects of International Cooperation and Exchanges (81820108019), Beijing Tianjin and Hebei Special Foundation (19JCZDJC63900), the Capital Clinical Feature Research Project (Z171100001017158), Big Data Program of Chinese PLA General Hospital (2017MBD-008), and Translational Medicine Program of Chinese PLA general hospital (2017TM- 003).

## Conflict of Interest

The authors declare that the research was conducted in the absence of any commercial or financial relationships that could be construed as a potential conflict of interest.

## Publisher's Note

All claims expressed in this article are solely those of the authors and do not necessarily represent those of their affiliated organizations, or those of the publisher, the editors and the reviewers. Any product that may be evaluated in this article, or claim that may be made by its manufacturer, is not guaranteed or endorsed by the publisher.
